# Effectiveness of Remote Fetal Monitoring on Maternal-Fetal Outcomes: Systematic Review and Meta-Analysis

**DOI:** 10.2196/41508

**Published:** 2023-02-22

**Authors:** Suya Li, Qing Yang, Shuya Niu, Yu Liu

**Affiliations:** 1 Nursing Department Tongji Hospital, Tongji Medical College HuaZhong University of Science and Technology Wuhan China; 2 Zhongnan Hospital of Wuhan University Wuhan China

**Keywords:** remote fetal monitoring, maternal outcomes, fetal outcomes, review

## Abstract

**Background:**

To solve the disadvantages of traditional fetal monitoring such as time-consuming, cumbersome steps and low coverage, it is paramount to develop remote fetal monitoring. Remote fetal monitoring expands time and space, which is expected to popularize fetal monitoring in remote areas with the low availability of health services. Pregnant women can transmit fetal monitoring data from remote monitoring terminals to the central monitoring station so that doctors can interpret it remotely and detect fetal hypoxia in time. Fetal monitoring involving remote technology has also been carried out, but with some conflicting results.

**Objective:**

The review aimed to (1) examine the efficacy of remote fetal monitoring in improving maternal-fetal outcomes and (2) identify research gaps in the field to make recommendations for future research.

**Methods:**

We did a systematic literature search with PubMed, Cochrane Library, Web of Science, Embase, MEDLINE, CINAHL, ProQuest Dissertations and Theses Global, ClinicalTrials.gov, and Open Grey in March 2022. Randomized controlled trials or quasi-experimental trials of remote fetal monitoring were identified. Two reviewers independently searched articles, extracted data, and assessed each study. Primary outcomes (maternal-fetal outcomes) and secondary outcomes (health care usage) were presented as relative risks or mean difference. The review was registered on PROSPERO as CRD42020165038.

**Results:**

Of the 9337 retrieved literature, 9 studies were included in the systematic review and meta-analysis (n=1128). Compared with a control group, remote fetal monitoring reduced the risk of neonatal asphyxia (risk ratio 0.66, 95% CI 0.45-0.97; *P*=.04), with a low heterogeneity of 24%. Other maternal-fetal outcomes did not differ significantly between remote fetal monitoring and routine fetal monitoring, such as cesarean section (*P*=.21; *I*^2^=0%), induced labor (*P*=.50; *I*^2^=0%), instrumental vaginal birth (*P*=.45; *I*^2^=0%), spontaneous delivery (*P*=.85; *I*^2^=0%), gestational weeks at delivery (*P*=.35; *I*^2^=0%), premature delivery (*P*=.47; *I*^2^=0%), and low birth weight (*P*=.71; *I*^2^=0%). Only 2 studies performed a cost analysis, stating that remote fetal monitoring can contribute to reductions in health care costs when compared with conventional care. In addition, remote fetal monitoring might affect the number of visits and duration in the hospital, but it was not possible to draw definite conclusions about the effects due to the limited number of studies.

**Conclusions:**

Remote fetal monitoring seems to reduce the incidence of neonatal asphyxia and health care costs compared with routine fetal monitoring. To strengthen the claims on the efficacy of remote fetal monitoring, further well-designed studies are necessary, especially in high-risk pregnant women, such as pregnant women with diabetes, pregnant women with hypertension, and so forth.

## Introduction

Fetal safety has always been a top priority for perinatal care. According to the World Health Organization, as of 2019, there were an estimated 2 million stillbirths, most of which can be prevented by safe and quality care, timely emergency care, and accurate recording [1]. Fetal monitoring is the primary means of monitoring to assess fetal safety and contributes to reducing the risk of stillbirth by detecting fetal hypoxia as early as possible [2,3]. Previous studies have repeatedly demonstrated the clinical value of fetal monitoring in reducing adverse perinatal outcomes (eg, neonatal cerebral palsy, hypoxic-ischemic encephalopathy, or stillbirth) [4,5].

Traditional antenatal care is resource intensive and not friendly to underserved settings. Beyond that, routine prenatal monitoring is only suitable for hospital settings, which means that pregnant women require regular outpatient follow-up [6]. Recurrent outpatient visits also pose additional travel risks (eg, falls, collisions, and bumps), especially for high-risk pregnant women. Telemedicine refers to the long-distance transmission of medical information between medical workers and patients through telecommunication technology [7], which has many potential advantages such as reducing outpatient time, alleviating the shortage of medical resources, reducing transportation costs and medical costs, and so forth [8-10]. Remote monitoring using telephones, websites, portable devices, and so forth during pregnancy is becoming more and more popular [11,12].

Systematic reviews have demonstrated the feasibility and superiority of telemedicine in obstetrics [13], focusing on blood pressure (BP) management [14,15], blood glucose management [16], and weight management [17] during pregnancy. However, we are not yet clear about the benefits or dangers of remote fetal monitoring. The primary objective of this systematic review was to assess the effectiveness of remote fetal monitoring for improving maternal-fetal outcomes. In addition, we also sought to analyze the cost-effectiveness of remote fetal monitoring compared to conventional prenatal monitoring.

## Methods

### Reporting Standards

This systematic review and meta-analysis was carried out according to the Preferred Reporting Items for Systematic Reviews and Meta-Analyses guidelines of 2009 [18] and was registered on PROSPERO as CRD42020165038.

### Literature Retrieval

In total, 9 web-based databases were searched in March 2022, including PubMed (January 1966-March 2022), Cochrane Library (January 1947-March 2022), Web of Science (January 1990-Mar 2022), Embase (January 1974-March 2022), MEDLINE (January 1950-March 2022), CINAHL (January 1982-March 2022), ProQuest Dissertations and Theses Global (January 1899-March 2022), ClinicalTrials.gov (January 1997-March 2022), and Open Grey (January 1980-March 2022). Search terms generated from the inspection of relevant papers were wielded to search for eligible studies, such as fetal, remote, telemetry, monitor, and so forth. The full search strategy was available in Multimedia Appendix 1 and was rerun before the final analysis.

### Inclusion Criteria

Studies were considered eligible if they simultaneously met the following criteria: (1) pregnant women; (2) randomized controlled trials (RCTs) or quasi-experimental trials; (3) fetal monitoring data were transmitted to the central monitoring station by remote monitoring terminals; and (4) outcomes included at least 1 maternal-fetal outcome or health resource usage. There were no restrictions on language, nationality, or publication status.

### Exclusion Criteria

Studies meeting any of the following criteria were excluded: (1) no control group in the study; (2) comparative studies of 2 or more remote monitoring technologies; and (3) the full text was still unavailable after contacting the original authors. Studies were not excluded due to monitoring settings (hospital, home, community setting, or mixed).

### Outcome Measures

The primary outcomes were maternal-fetal outcomes (cesarean section, induced labor or miscarriage, instrumental vaginal birth, spontaneous delivery, gestational weeks at delivery, premature delivery, birth weight, and so on). The secondary outcomes were health care usage, which was assessed by on-site appointments, home visits, duration in the hospital, prenatal costs, and so on.

### Study Selection

A 3-step screening identified articles that met the inclusion and exclusion criteria were literature retrieval, preliminary screening (title and abstract), and full-text screening. Literature retrieval was conducted by 2 investigators. All searched articles were uploaded into the reference management tool of EndNote. Articles with the same author, year, title, and so on were identified and removed by EndNote. Subsequently, 2 independent investigators (SYL and QY) selected all articles by evaluating the title and abstract after the removal of duplicates. Finally, the same 2 investigators (SYL and QY) identified the ultimately eligible articles by screening independently the full text according to the inclusion and exclusion criteria. In addition, the first author (SYL) hand-searched the references of the ultimately included literature to identify further publications. Any discrepancies and disagreements were finally resolved by consultation with a third reviewer (YL). We also contacted the original authors for verification if there were any uncertain technical types.

### Data Extraction

Data from included studies were extracted by SYL and then cross-checked by another author (QY). A standardized data extraction form was designed by the research team and included the following data: (1) basic information of included studies (first author, year of publication, country, and study design); (2) characteristics of participants (maternal age, gestational weeks, sample size, and attrition rate); (3) characteristics of interventions (trial settings, duration of the intervention, monitoring personnel, monitoring content, feedback types, and technical support); and (4) outcomes measurement (maternal-fetal outcomes and health care usage). For insufficient data, we contacted the original authors via email. The standardized data extraction form was available in Multimedia Appendix 2.

### Quality Assessment

Independently, the quality of eligible studies was assessed by 2 reviewers (SYL and QY) according to the Cochrane Risk of Bias Tool [19], which consisted of 7 items (random sequence generation, allocation concealment, blinding of participants and personnel, blinding of outcome assessment, incomplete outcome data, selective reporting, and other bias) with the responses of “low risk,” “high risk,” and “unclear risk.” The research was considered high quality with a low risk score on at least 4 domains, which must include 3 key domains (random sequence generation, allocation concealment, and incomplete outcome data) [20]. Consensuses between 2 investigators (SYL and QY) were reached by discussion with a third reviewer (YL).

### Data Synthesis and Statistical Analysis

Quantitative analysis of included studies was carried out in Review Manager (RevMan) software (version 5.4). Continuous variables were presented as mean difference (MD), and dichotomous variables were described as risk ratio (RR) with a 95% CI. The statistical heterogeneity of selected studies was assessed by the chi-square test combined with *I*^2^. Heterogeneity was divided into nonsignificant heterogeneity (*I*^2^ ranging from 0% to 40%), moderate heterogeneity (*I*^2^ ranging from 30% to 60%), substantial heterogeneity (*I*^2^ ranging from 50% to 90%) and considerable heterogeneity (*I*^2^ ranging from 75% to 100%) [19]. When *I*^2^<40%, the fixed-effects model was adopted; otherwise, a random effect model was considered. In addition, sensitivity analysis and subgroup analysis were used to explore the sources of heterogeneity if needed.

## Results

### Study Selection

**Figure 1:**
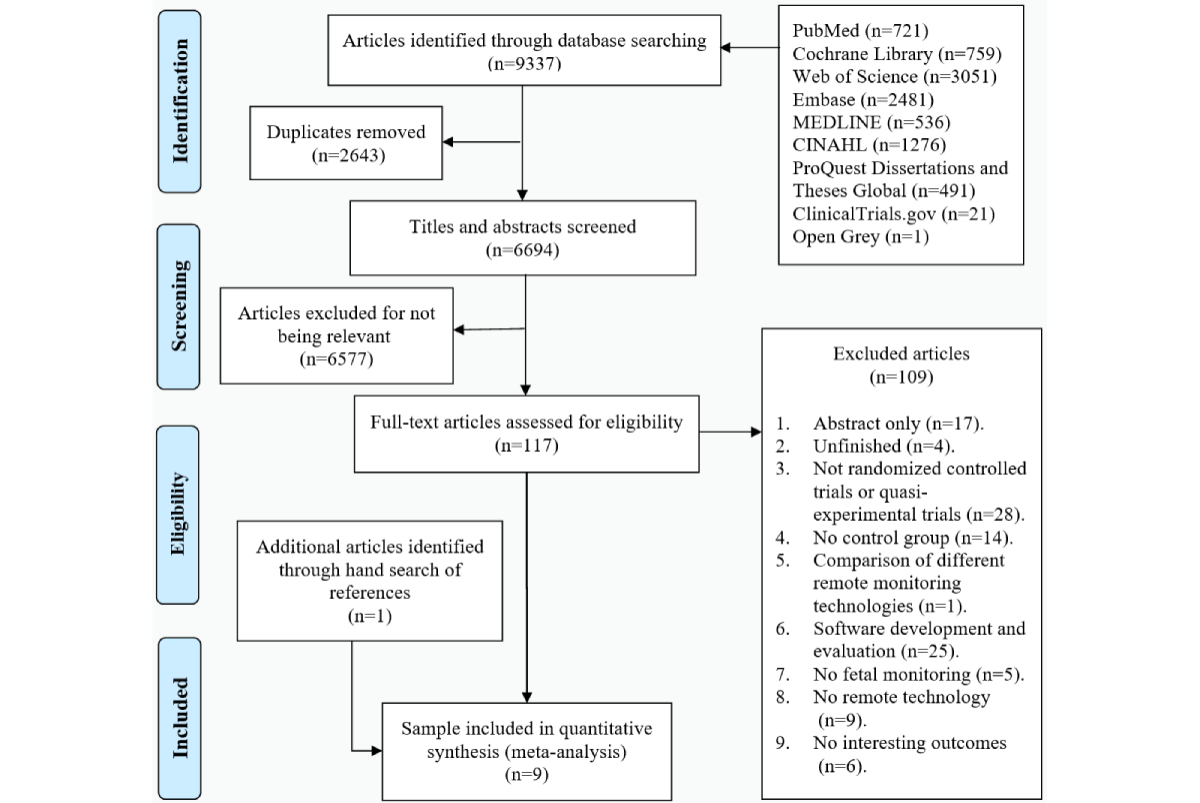
Flow diagram of study selection.

A total of 9337 studies were initially retrieved by searching 9 databases. After the 3-step screening, 8 studies met the inclusion and exclusion criteria. From a manual search of related references, 1 additional study was included. Finally, 9 RCTs were included in the systematic review and meta-analysis. The results of 1 study were published in 2 articles [21,22]. The detailed flow diagram of study selection is shown in Figure 1.

### Study Characteristics

The characteristics of 9 RCTs are outlined in Table 1, involving 1128 participants from 6 countries. Seven studies were from developed countries (1 from the United States [23], 3 from the United Kingdom [24-26], 2 from the Netherlands [21,22], and 1 from Finland [27]). Only 2 studies were performed in developing countries (1 from China [28] and 1 from Mexico [29]). Eight of the screened studies were monocentric, and 1 was multicenter [26]. On the duration of interventions, 7 studies were carried out in the prenatal period [21-23,25,26,28,29], and 2 studies were conducted during labor [24,27]. In terms of participants, most of the included studies recruited high-risk pregnant women [21,22,25,26], and the remaining studies recruited low-risk pregnant women [23], late pregnant women [28], and pregnant women facing labor [24,25], respectively. The pooled mean age of pregnant women was 29.28 (SD 5.03) years in 6 RCTs [21-23,25-27].

**Table 1:** Characteristics of included studies.

Author, year, country	Study design	Participants	Duration	Sample, N	Attrition rate (%)	Major characterization	Major results
Butler Tobah et al, 2019, United States [23]	2-arm RCT,^a^ monocentric	Low-risk pregnancies (IG：29.5±3.3 years; CG：29.7±3.6 years)	<13 weeks of gestation to deliver	IG^b^: N=134; CG^c^: N=133	11	IG: OB Nest care (8 on-site appointments, 6 remote visits via phone or web-based communication) CG: usual care (12 prescheduled prenatal clinic appointments)	Pregnancy outcomes (cesarean, delivery, miscarriage, and preterm delivery); neonatal outcomes (low birth weight, and neonatal asphyxia); health care usage (on-site appointments, remote visits, and inpatient days)
Wang et al, 2019, China [28]	2-arm RCT, monocentric	Late pregnancies (IG：22-40 years; CG：22-38 years)	36-41 weeks of gestation to deliver	IG: N=80; CG: N=80	0	IG: remote FHR^d^ monitoring (3-4 times daily); CG: own fetal movement count (3 times daily) and routine outpatient FHR monitoring	Neonatal outcomes (neonatal asphyxia and nonstress test）
Tapia-Conyer et al, 2015, Mexico [29]	2-arm RCT, monocentric	High-risk pregnancies (<19 or >35 years)	27-29 weeks of gestation to deliver	IG: N=74; CG: N=79	12	IG: wireless maternal-fetal monitoring (1- to 2-week intervals); CG: conventional care (standard midwifery visits)	Pregnancy outcomes (preterm, preeclampsia, and eclampsia); neonatal outcomes (low birth weight); adherence
Dawson et al, 1999, United Kingdom [26]	2-arm RCT, multicenter	High-risk pregnancies (IG：25.7 ± 5.0 years; CG：27.2 ± 6.3 years)	12 weeks of gestation to deliver	IG: N=43; CG: N=38	0	IG: domiciliary monitoring daily via DFM^e^ system; CG: conventional care (standard midwifery visits)	Pregnancy outcomes (weeks of gestation at delivery, spontaneous delivery, cesarean delivery, operative vaginal delivery, and induced labor); neonatal outcomes (neonatal asphyxia); health care usage (on-site appointments, home visits, inpatient days, and cost-effectiveness)
Birnie et al, 1997, the Netherlands [21]	2-arm RCT, monocentric	High-risk pregnancies (IG：29.6±5.8 years; CG：30.9±5.8 years)	32-43 weeks of gestation to deliver	IG: N=76; CG: N=74	0	IG: domiciliary monitoring daily via portable cardiotocography; CG: in-hospital monitoring daily	Pregnancy outcomes (weeks of gestation at delivery, cesarean delivery, and induced labor); neonatal outcomes (birth weight); health care usage (inpatient days and cost-effectiveness)
Monincx et al, 1997, the Netherlands [22]	2-arm RCT, monocentric	High-risk pregnancies (IG：29.6±5.8 years; CG：30.9±5.8 years)	32-43 weeks of gestation to deliver	IG: N=76; CG: N=74	0	IG: domiciliary monitoring daily via portable cardiotocography; CG: in-hospital monitoring daily	Pregnancy outcomes (spontaneous delivery, operative vaginal delivery, and perinatal mortality); neonatal outcomes (neonatal asphyxia and neurological optimality scores)
Dawson et al, 1989, United Kingdom [25]	2-arm RCT, monocentric	High-risk pregnancies (IG：28.78±5.85 years; CG：26.06±3.51 years)	26-41 weeks of gestation to deliver	IG: N=40; CG: N=17	5	IG: domiciliary monitoring daily via DFM system; CG: conventional hospital care	Pregnancy outcomes (weeks of gestation at delivery, cesarean delivery, and induced labor)
Calvert et al, 1982, United Kingdom [24]	3-arm RCT, monocentric	Patients facing labor (≤37 weeks of gestation)	During labor	IG: N=100; CG: N=100	0	IG: remote monitor cardiotocography (patients could get out of bed to walk or sit); CG: conventional bedside cardiotocography	Pregnancy outcomes (spontaneous delivery, cesarean delivery, and operative vaginal delivery); Neonatal outcomes (neonatal asphyxia)
Haukkamaa et al, 1982, Finland [27]	2-arm RCT, monocentric	Patients facing labor (IG：28.35±3.75 years; CG：28.1±3.7 years)	During labor	IG: N=31; CG: N=29	0	IG: FHR monitored by telemetry (patients were encouraged to sit or walk); CG: FHR monitored by conventional cardiotocography	Pregnancy outcomes (cesarean delivery, operative vaginal delivery, and induced labor)

^a^RCT: randomized controlled trial.

^b^IG: intervention group.

^c^CG: control group.

^d^FHR: fetal heart rate.

^e^DFM: domiciliary fetal monitoring.

### Characteristics of Interventions

The characteristics of interventions are described in Table 2. Most of the included studies were undertaken at home [21-23,25,26,28], with 3 exceptions occurring in rural clinics [29] and hospitals [24,27]. Pregnant women in the control groups received “conventional care,” including routine outpatient monitoring, in-hospital monitoring, or conventional bedside cardiotocography. Pregnant women in the intervention groups received remote fetal monitoring with web, Bluetooth, or telephone. Of the included studies, 5 RCTs only supervised fetal heart rate [24-28], and the remaining 4 RCTs monitored extra BP [21-23,29], blood glucose [29], height [29], weight [29], or temperature [21,22].

The frequency of fetal monitoring and guidance varied among the included studies as did the form of feedback. Due to the different stages of pregnancy, the frequency of fetal monitoring ranged from 3 to 4 times daily to biweekly. There were many ways to achieve one-to-one, personalized, and exclusive guidance, including phone visits, on-site appointments, or family visits. In addition, 2 other studies, which occurred during labor, used the obstetrical telemetry system to remotely monitor the fetus in real time [24,27]. During the birth process, the pregnant women in the conventional group were nursed in bed, whereas those with telemetry equipment were encouraged to get out of bed to walk or sit on a chair.

**Table 2:** Characteristics of interventions.

Author, year, country	Monitoring personnel	Monitoring locus	Monitoring content	Feedback	Technical support
Butler Tobah et al, 2019, United States [23]	Patient, nurse, and obstetrician	Domiciliary	FHR,^a^ BP^b^	Transmission of data via a phone or the institution’s electronic medical record system Personalized guidance by telephone visits or on-site appointments	Home digital sphygmomanometer, handheld fetal Doppler, and patient web portal
Wang et al 2019, China [28]	Patient and obstetrician	Domiciliary	FHR	Transmission of data via phone Personalized guidance via telephone if necessary	Portable intelligent medical terminal system
Tapia-Conyer et al, 2015, Mexico [29]	Nurse and obstetrician	Rural clinics	FHR, BP, blood glucose, height, and weight	Transmission of data through a Bluetooth interface and web access Personalized consultations via fetal monitoring visits	MiBebe fetal remote monitor prototype, Bluetooth, and patient web portal
Dawson et al 1999, United Kingdom [26]	Patient, community midwife	Domiciliary	FHR	Transmission of data via telephone using modems Personalized surveillance and care for each pregnant woman	DFM^c^ system
Birnie et al 1997, the Netherlands [21]	Investigator, midwife, and physician	Domiciliary	FHR, BP, and temperature	Transmission of data via telephone Personalized consultations via telephone if necessary	Portable cardiotocography and public telephone network
Monincx et al 1997, the Netherlands [22]	Investigator, midwife, and physician	Domiciliary	FHR, BP, and temperature	Transmission of data via telephone Personalized consultations via telephone if necessary	Portable cardiotocography and public telephone network
Dawson et al 1989, United Kingdom [25]	Patient, midwife	Domiciliary	FHR	Transmission of data via telephone fetal monitoring systems Personalized guidance via regular family visits	DFM system
Calvert et al 1982, United Kingdom [24]	Midwife	Hospital	FHR	Transmission of data via an obstetrical telemetry system	Obstetrical telemetry system
Haukkamaa et al 1982, Finland [27]	Midwife	Hospital	FHR	Transmission of data via an obstetrical telemetry system	Obstetrical telemetry system

^a^FHR: fetal heart rate.

^b^BP: blood pressure.

^c^DFM: domiciliary fetal monitoring.

### Risk of Bias

**Figure 2:**
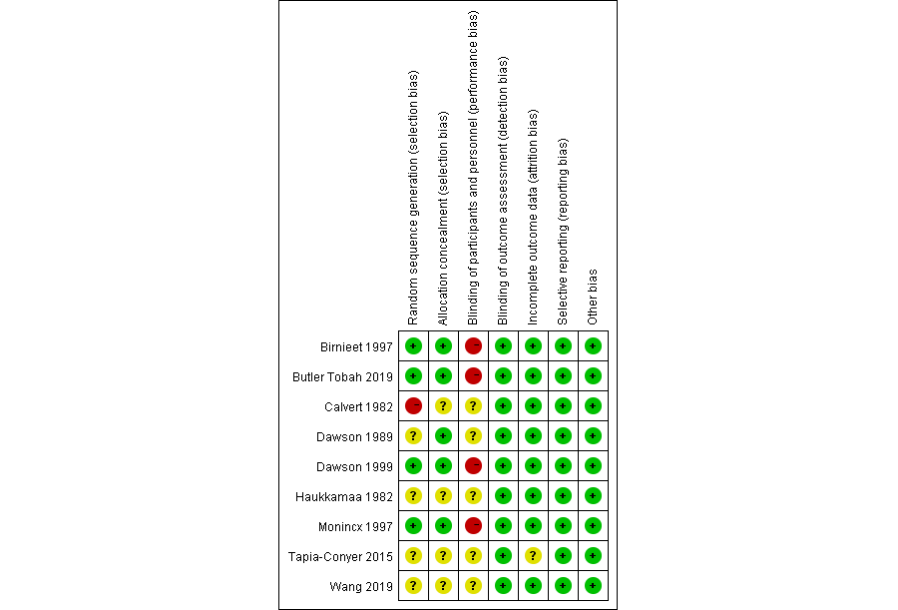
Risk of bias in each study.

**Figure 3:**
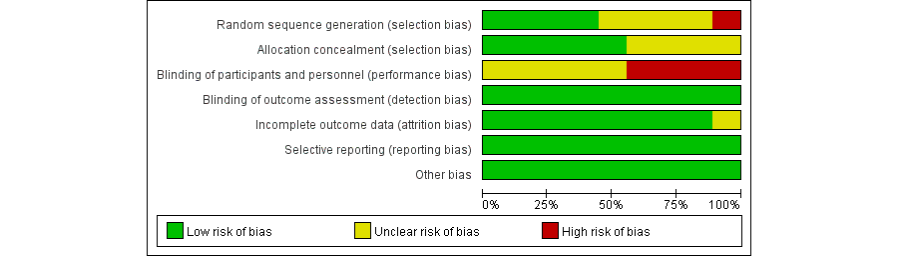
Overall risk of each type of bias.

Overall, the quality of included studies was moderate, 4 of which (44%) were high-quality research [21-23,26]. The studies showed the main bias in the blinding of participants and personnel, which might be caused by the nature of interventions. In addition, 1 study (11%) showed a high risk of bias for random sequence generation because of grouping according to the hospital number [24]. Fortunately, all outcomes were obtained from medical records, so the outcome assessment would not be influenced by the lack of blinding. Based on the above reasons, the blinding of outcome assessment of included studies was assessed as “low risk of bias.” Three RCTs (22%) reported clear data loss, with attrition of 11% [23], 12% [29], and 5% [25], respectively. One of the studies had a relatively large difference in attrition between the groups (20% and 4%, respectively), and it was unclear whether the loss to follow-up varied [29]. Three studies (22%) used intention-to-analysis [21-23] (Figures 2 and 3).

### Synthesis of Results

The review extracted 8 maternal-fetal outcomes and the pooled analyses are presented in Table 3.

**Table 3:** Effect estimates of 8 outcomes.

Outcomes	Studies, n	Participants, n	Statistical methods	Effect estimates
Cesarean section	6	815	Risk ratio (M-H,^a^ fixed, 95% CI)	0.81 (0.59 to 1.12)
Neonatal asphyxia	5	859	Risk ratio (M-H, fixed, 95% CI)	0.66 (0.45 to 0.97)^b^
Instrumental vaginal birth	4	492	Risk ratio (M-H, fixed, 95% CI)	1.21 (0.74 to 1.98)
Induced labor	4	348	Risk ratio (M-H, fixed, 95% CI)	0.90 (0.66 to 1.22)
Spontaneous delivery	3	432	Risk ratio (M-H, fixed, 95% CI)	0.99 (0.89 to 1.10)
Gestational weeks at delivery	3	288	Mean difference (IV,^c^ fixed, 95% CI)	−0.28 (−0.86 to 0.30)
Premature delivery	2	420	Risk ratio (M-H, fixed, 95% CI)	0.80 (0.44 to 1.46)
Low birth weight	2	420	Risk ratio (M-H, fixed, 95% CI)	1.20 (0.45 to 3.20)

^a^M-H: Mantel-Haenszel.

^b^Statistically significant at *P*=.04 level.

^c^IV: inverse variance.

### Maternal Outcomes

**Figure 4:**
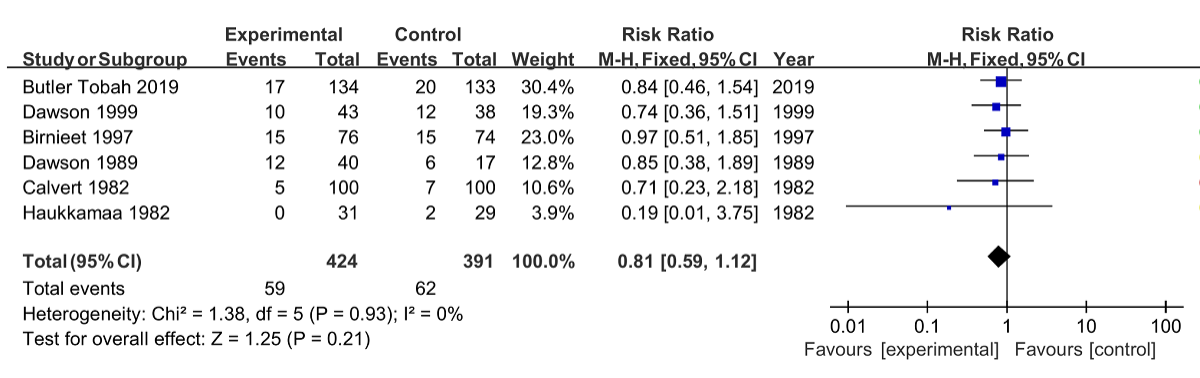
Forest plot of cesarean section.

Cesarean section was the most assessed in the included studies, involving 815 pregnant women from 6 RCTs [21,23-27]. Under the fixed effect model, the pooled results showed a nonsignificant difference between the intervention group and the control group (RR 0.81, 95% CI 0.59-1.12; *P*=.21), without any heterogeneity (*I*^2^=0%; *P*=.93; Figure 4).

**Figure 5:**
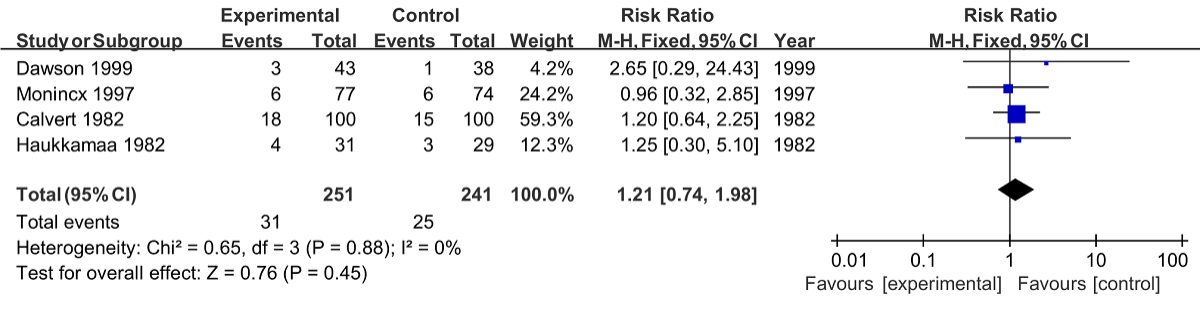
Forest plot of instrumental vaginal birth.

Instrumental vaginal birth was mentioned in 4 studies involving 492 pregnant women [22,24,26,27]. There was no evidence of heterogeneity when pooling the 4 studies (*I*^2^=0%; *P*=.88). With a fixed effect model, the prevalence of instrumental vaginal birth did not significantly differ between the remote monitoring group and the routine monitoring group (RR 1.21, 95% CI 0.74-1.98; *P*=.45; Figure 5).

**Figure 6:**
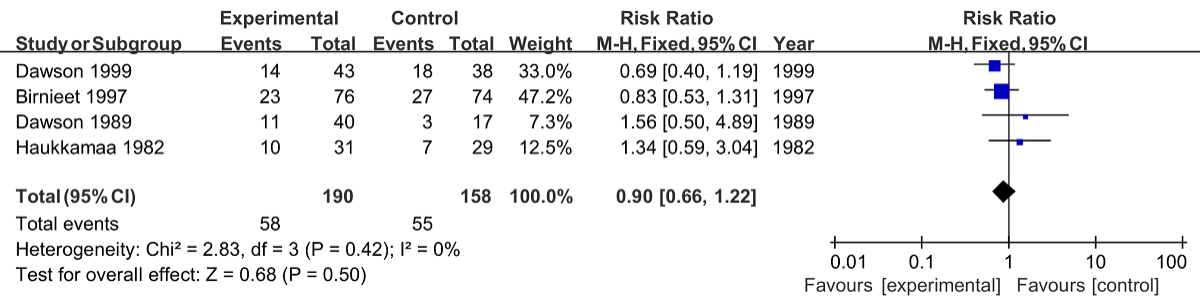
Forest plot of induced labor.

Four RCTs (n=348) reported induced labor with an overall rate of 32% [21,25-27]. Moreover, no significant difference (RR 0.90, 95% CI 0.66-1.22; *P*=.50) between groups and the heterogeneity (*I*^2^=0%; *P*=.42) in pooling 4 studies was demonstrated (Figure 6).

**Figure 7:**
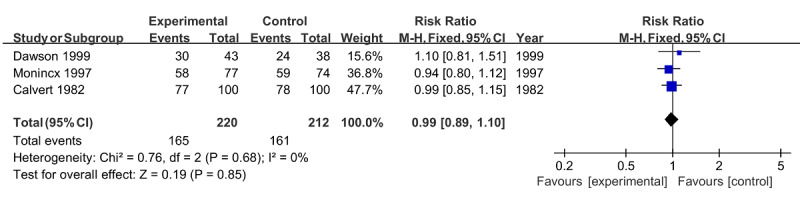
Forest plot of spontaneous delivery.

**Figure 8:**
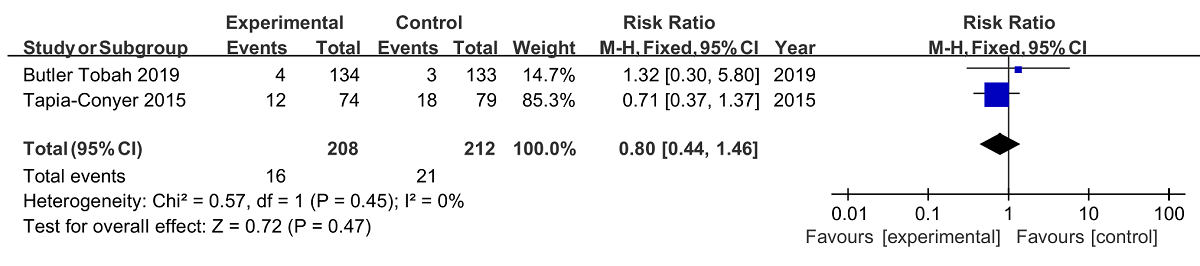
Forest plot of premature delivery.

**Figure 9:**
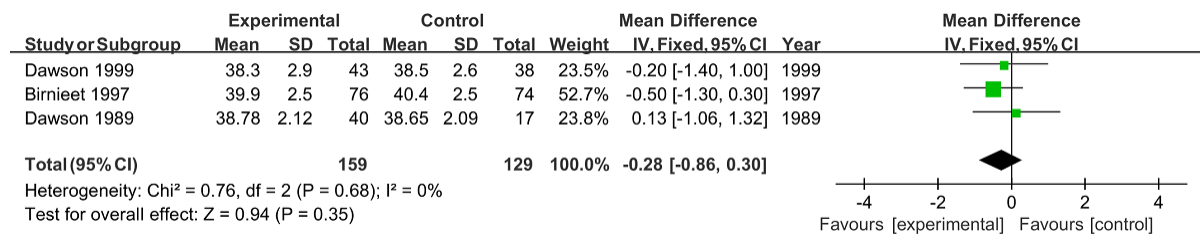
Forest plot of gestational weeks at delivery.

Similarly, no significant difference was found in the risk of spontaneous delivery (RR 0.99, 95% CI 0.89‐1.10; *P*=.85) [22,24,26] or premature delivery (RR 0.80, 95% CI 0.44‐1.46; *P*=.47) [23,29], both with no heterogeneity (*I*^2^=0%; *P*=.68 and *P*=.45, respectively; Figures 7 and 8). For gestational weeks at delivery, the overall effect of 3 studies [21,25,26] was also insignificant (MD −0.28, 95% CI −0.86 to 0.30; *P*=.35) in the absence of heterogeneity (*I*^2^=0%; *P*=.68; Figure 9).

### Outcomes of Infants

**Figure 10:**
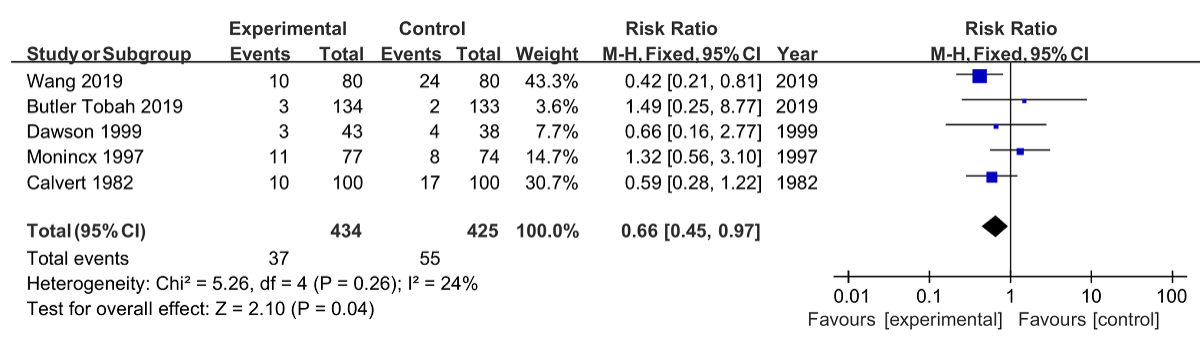
Forest plot of neonatal asphyxia.

Five studies (n=859) compared the incidence of neonatal asphyxia between the intervention group and the control groups, with an overall prevalence of 11% [22-24,26,28]. Furthermore, the overall effect of neonatal asphyxia was significant, and the combined risk ratio was 0.66 (95% CI 0.45-0.97; *P*=.04) with an acceptable heterogeneity across studies (*I*^2^=24%; *P*=.26; Figure 10).

**Figure 11:**
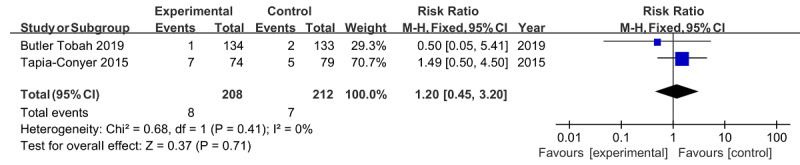
Forest plot of low birth weight.

For low birth weight, the pooled results of 2 studies involving 420 newborns showed that no significant difference was discovered between the intervention group and the control group (RR 1.20, 95% CI 0.45-3.20; *P*=.71), without any heterogeneity (*I*^2^=0%; *P*=.41; Figure 11) [23,29].

### Health Care Usage

The outcomes of health care usage were investigated in 3 studies [21,23,26], involving the number of on-site appointments or home visits, duration in hospital, medical cost, and so on. However, none of them was suitable for meta-analysis due to heterogeneity of evaluation methods and assessment timing or to a lack of sufficient data.

Butler Tobah et al [23] reported that compared with conventional nursing, the number of on-site appointments with clinicians and nurses decreased significantly in the intervention group (11.25 vs 14.69 visits; *P*<.01), while the duration of time spent on coordinating care and connected care appointments by phone or on the internet was higher in the intervention group (401.20 vs 167.10 minutes per woman; *P*<.01). Similarly, Dawson et al [26] also reported that the remote group received more home visits (3.7 vs 1.4 visits; *P*=.002) and longer home visits (33.5 vs 12.8 minutes per visit; *P*<.001). There was no significant difference in the number of antenatal clinic visits between the 2 groups (2.4 vs 3.2 visits; *P*=.11) [26]. For antenatal inpatient days, Dawson et al [26] found there were no significant differences between the 2 groups (3.58 vs 5.13 days; *P*=.12), whereas Birnie et al [21] reported longer hospital stays in the control group (1 vs 7 days; *P*<.001). Furthermore, no significant differences in hospital length of stay after delivery [21,23] were observed across groups.

Two studies reported cost-effectiveness [21,26]. Birnie et al [21] indicated that domiciliary monitoring had lower prenatal costs than in-hospital monitoring (US $1521 vs US $3558 per woman; *P*<.001), mainly focusing on nursing care, domiciliary monitoring, and informal family care. Dawson et al [26] also supported that the total cost of domiciliary care was €223.83 (US $239.89 in 2023) per woman less than that of conventional care, consisting of community midwife (time and travel), home monitoring equipment (capital cost and maintenance), lost productive output (women and partners), and antenatal clinic attendances (visits, ultrasound scans, and antenatal inpatient care) [26].

## Discussion

### Principal Findings

As far as we know, this is the first article to quantitatively analyze the effects of remote fetal monitoring. The systematic review and meta-analysis highlighted that remote fetal monitoring significantly reduced the risk of neonatal asphyxia by 34%. Beyond that, remote fetal monitoring was also beneficial for reducing prenatal costs, which showed some potential for greater cost-effectiveness.

### Comparison With Prior Studies

In previous reviews, the superiority of obstetric remote monitoring has also been repeatedly emphasized because of real-time, periodic, and remote monitoring [3,30,31]. By integrating 14 studies involving blood glucose, fetal heart rate, and uterine activity, Lanssens et al found that remote monitoring reduced low neonatal birth weight and neonatal intensive care unit admissions, as well as prolonged gestational age [31]. Likewise, a recent systematic review, focusing on obstetric remote monitoring of BP, uterine contractions, weight, heart rate, and so forth also supported that telemonitoring during pregnancy had great potential for promoting better pregnancy outcomes [3]. However, due to limited research on prenatal remote monitoring, no further quantitative analysis was carried out in the above reviews.

This systematic review and meta-analysis, the first to focus remote monitoring on the fetus, revealed that remote fetal monitoring reduced the risk of neonatal asphyxia by 34%. Remote fetal monitoring can identify signs of fetal hypoxia in time by monitoring wherever and whenever, which is essential to reduce neonatal asphyxia, especially in high-risk pregnant women [32]. In terms of cost-effectiveness, only 2 RCTs out of 9 studies reported cost-effectiveness [21,26]. Both demonstrated that remote monitoring significantly reduced prenatal costs, which was consistent with previous studies [31,33,34]. In Lanssens’ [31] review, 2 retrospective studies found that remote monitoring significantly reduced health care costs. In the studies reviewed, cost analysis focused on health care costs, patient costs, caregiver costs, and productivity costs. Remote fetal monitoring had additional equipment costs and maintenance costs, but in the long run, it saved much more than that, such as time costs, travel costs, or outpatient costs.

In addition, the disadvantages of remote fetal monitoring remained controversial, such as whether additional cesarean sections would be added. In this regard, this meta-analysis covering 9 studies found no consistent evidence of adverse effects on maternal and infant outcomes, with a small heterogeneity ranging from 0% to 24%. This might be related to accurate guidance from midwives or obstetricians on the remote monitoring team. Nonetheless, a recent review in 2019 evaluated information involving decreased fetal movement in 24 mobile applications, revealing that the information varied widely and lacked evidence-based clinical advice [35]. Accurate information about fetal movement is essential for improving maternal and infant outcomes. Therefore, it is recommended that health care personnel cooperate with software developers to jointly develop high-quality prenatal education tools, which will help to promote more pregnant women to obtain timely and accurate guidance.

Notably, in the current systematic review and meta-analysis, 7 of the 9 studies were carried out in developed countries, which were inseparable from the rich medical resources and advanced medical technologies of developed countries. The latest global figures showed that in 2020, there were 26 and 17 deaths per 1000 live births in low- and middle-income countries (LMICs), respectively. However, in high-income countries, the rate only stood at 3 per 1000 [36]. Given the higher perinatal mortality rate, the need for remote fetal monitoring in developing countries may be more urgent. Furthermore, a recent review focused on LMICs concluded that mobile technology can overcome economic and geographic barriers by transmitting clinical information collected using low-cost devices, thereby increasing the perinatal care coverage of LMICs [5]. It can be argued that remote fetal monitoring supported by mobile technology appears to have greater potential in LMICs, where antenatal care services need to be improved. Therefore, we encourage remote fetal monitoring in LMICs to alleviate the shortage of medical resources and further complement the benefits of remote fetal monitoring.

### Suggestions for Clinical Practice

This systematic review has demonstrated that remote fetal monitoring has a significant effect on improving maternal and infant outcomes, but this does not mean that remote fetal monitoring can replace face-to-face communication between doctors and patients, which is necessary for shared decision-making. Remote monitoring breaks through the barriers of time and distance, so it is reasonable as an effective complement to traditional outpatient monitoring [37]. Especially during the COVID-19 pandemic, pregnant women, as a high-risk group, should not gather in outpatient clinics for a long time. At this time, remote fetal monitoring not only realizes noncontact medical services but also ensures the safety of mothers and babies. Unfortunately, remote fetal monitoring is rarely implemented in developing countries, especially in areas with limited medical resources [3]. Therefore, the development and implementation of remote monitoring technology urgently need to be put on the agenda. Aside from the technical issues, another concern of remote fetal monitoring is that authentication rules, reimbursement policies, data security, legal responsibilities, and so forth are not yet clear [38]. Although remote fetal monitoring has not yet shown adverse consequences, it is still necessary to conduct relevant research cautiously in combination with the local medical level.

### Limitations

There were some limitations worth noting. The diversity of pregnant women in the current systematic review was the major limitation, involving low-risk pregnancies, high-risk pregnancies, late pregnancies, and patients facing labor. Future research can continue to explore which types of pregnant women are more suitable for remote fetal monitoring. In addition, several RCTs included in this meta-analysis were relatively old, which might limit the direct applicability of the evidence to current clinical practice. Finally, due to the limited literature, it was difficult to quantitatively analyze the efficacy of remote fetal monitoring in health resource usage. Future studies are expected to assess the cost-effectiveness of remote fetal monitoring, including implementation costs (technology costs, medical costs, etc), intervention costs (patient resource costs, commuting costs, etc), and downstream costs (productivity costs, future costs, etc) [39]. Likewise, the number of consultations, length of hospital stay, and patient compliance or satisfaction cannot be ignored and need to be explored further.

### Conclusions

The present systematic review and meta-analysis of 9 studies highlighted that remote fetal monitoring had a favorable effect on reducing neonatal asphyxia. Remote fetal monitoring has not yet found hidden dangers, but more large-scale, multicenter, and high-quality studies are still expected to explore its safety and efficacy. At the same time, more research is also recommended to further carry out the cost analysis of remote fetal monitoring, which will help alleviate the huge medical expenses.

## Appendix

Multimedia Appendix 1 Search strategy.

Multimedia Appendix 2 Data extraction form.

## Abbreviations

BP: blood pressure
LMIC: low- and middle-income country
MD: mean difference
RCT: randomized controlled trial
RR: risk ratio
