# Supplementary material for: Effectiveness of Remote Fetal Monitoring on Maternal-Fetal Outcomes: Systematic Review and Meta-Analysis
Source: JMIR Mhealth Uhealth. 2023 Feb 22;11:e41508. doi: 10.2196/41508 (PMC9996419; doi:10.2196/41508)
Supplement: Multimedia Appendix 1 [file mhealth_v11i1e41508_app1.docx]

**Appendix 1: Search strategy**

1. tele* OR tech* OR electronic OR digital* OR automat* OR eHealth OR mHealth OR internet*
2. Cyberspace OR wireless OR online OR comput* OR phone OR mobile OR PDA OR Symbian OR ipad* OR Software OR app*
3. remote* OR telemetry OR home
4. pocket OR hand OR wear*
5. 1 OR 2 OR 3 OR 4
6. monitor* OR surveillance
7. fetal OR fetus
8. 5 AND 6 AND 7
