# Supplementary material for: Effectiveness of Remote Fetal Monitoring on Maternal-Fetal Outcomes: Systematic Review and Meta-Analysis
Source: JMIR Mhealth Uhealth. 2023 Feb 22;11:e41508. doi: 10.2196/41508 (PMC9996419; doi:10.2196/41508)
Supplement: Multimedia Appendix 2 [file mhealth_v11i1e41508_app2.docx]

**Appendix 2: Data Extraction Form**

| Trial ID | Extractor | Year of publication |
| --- | --- | --- |
| Title | | |
| First author | | |
| Country | | |

# Methods

|  | Descriptions as stated in report/paper | | Location in text or source |
| --- | --- | --- | --- |
| Design |  | |  |
| Trial settings |  | |  |
| Ethical approval | Yes No Unclear |  |  |
| Notes: | | | |

# Participants

|  | Descriptions as stated in report/paper | | Location in text or source |
| --- | --- | --- | --- |
| Population description |  | |  |
| Inclusion criteria |  | |  |
| Exclusion criteria |  | |  |
| Method of recruitment |  | |  |
| Informed consent obtained | Yes No Unclear |  |  |
| Sample size |  | |  |
| Baseline imbalances | Yes No Unclear |  |  |
| Attrition rate |  | |  |
| Maternal age |  | |  |
| Gestational weeks |  | |  |
| Other sociodemographic characteristics |  | |  |
| Notes: | | | |

# Intervention

|  | Description as stated in report/paper | Location in text or source |
| --- | --- | --- |
| Group name |  |  |
| Description |  |  |
| Duration of intervention |  |  |
| Monitoring personnel |  |  |
| Monitoring locus |  |  |
| Monitoring content |  |  |
| Feedback types |  |  |
| Technical support |  |  |
| Compliance |  |  |
| Notes: | | |

# Outcomes

| **Outcome Measures (Continuous)** | | Total participantsN = | | | | | |
| --- | --- | --- | --- | --- | --- | --- | --- |
|  |  | **Intervention group**  **n =** | | | Control groupn = | | |
|  |  | **total** | **mean** | **SD** | **total** | **mean** | **SD** |
|  | **Primary (maternal-fetal outcomes):** |  |  |  |  |  |  |
| 1 | Gestational weeks at delivery |  |  |  |  |  |  |
| 2 | Birth weight |  |  |  |  |  |  |
| 3 | Others |  |  |  |  |  |  |
|  | **Secondary (healthcare utilization):** |  |  |  |  |  |  |
| 1 | Duration in hospital |  |  |  |  |  |  |
| 2 | Prenatal costs |  |  |  |  |  |  |
| 3 | Others |  |  |  |  |  |  |

| **Outcome Measures (Dichotomous)** | | Total participants **N =** | | | |
| --- | --- | --- | --- | --- | --- |
|  |  | **Intervention group**  **n =** | | Control groupn = | |
|  |  | **events** | **total** | **events** | **total** |
|  | **Primary (maternal-fetal outcomes):** |  |  |  |  |
| 1 | Cesarean section |  |  |  |  |
| 2 | Premature delivery |  |  |  |  |
| 3 | Others |  |  |  |  |
|  | **Secondary (healthcare utilization):** |  |  |  |  |
| 1 | On-site appointments |  |  |  |  |
| 2 | Home visits |  |  |  |  |
| 3 | Others |  |  |  |  |

# Additional information requested

|  |
| --- |
